# Improving the Cost-Effectiveness of Visual Devices for the Control of Riverine Tsetse Flies, the Major Vectors of Human African Trypanosomiasis

**DOI:** 10.1371/journal.pntd.0001257

**Published:** 2011-08-02

**Authors:** Johan Esterhuizen, Jean Baptiste Rayaisse, Inaki Tirados, Serge Mpiana, Philippe Solano, Glyn A. Vale, Michael J. Lehane, Stephen J. Torr

**Affiliations:** 1 Vector Group, Liverpool School of Tropical Medicine, Liverpool, United Kingdom; 2 Centre International de Recherche et Développement sur l'Élevage en zone Subhumide (CIRDES), Bobo-Dioulasso, Burkina Faso; 3 Natural Resource Institute, University of Greenwich, Chatham, Kent, United Kingdom; 4 Laboratoire Vétérinaire Central de Kinshasa, Kinshasa, Democratic Republic of the Congo; 5 Institut de Recherche pour le Développement, UMR 177 IRD-CIRAD, Montpellier, France; 6 SACEMA, University of Stellenbosch, Stellenbosch, South Africa; Johns Hopkins Bloomberg School of Public Health, United States of America

## Abstract

Control of the Riverine (Palpalis) group of tsetse flies is normally achieved with stationary artificial devices such as traps or insecticide-treated targets. The efficiency of biconical traps (the standard control device), 1×1 m black targets and small 25×25 cm targets with flanking nets was compared using electrocuting sampling methods. The work was done on *Glossina tachinoides* and *G. palpalis gambiensis* (Burkina Faso), *G. fuscipes quanzensis* (Democratic Republic of Congo), *G. f. martinii* (Tanzania) and *G. f. fuscipes* (Kenya). The killing effectiveness (measured as the catch per m^2^ of cloth) for small targets plus flanking nets is 5.5–15X greater than for 1 m^2^ targets and 8.6–37.5X greater than for biconical traps. This has important implications for the costs of control of the Riverine group of tsetse vectors of sleeping sickness.

## Author Summary

Sleeping Sickness (Human African Trypanosomiasis) is a serious threat to health and development in sub-Saharan Africa. Currently there are no vaccines or prophylactic drugs available to prevent contraction of the disease. Consequently vector control is the only method of disease prevention. In many areas, especially those lacking high densities of cattle, the only control option for routine use against tsetse flies are insecticide-treated targets or biconical traps. However, these methods in their current form are often too expensive for routine use against the riverine tsetse species that are the major vectors of sleeping sickness. Our aim is to develop a more cost-effective device than those currently available. Working on four species of tsetse fly we have shown that a small 25×25 cm target with adjacent flanking net was up to 38x more cost-effective at killing tsetse flies than existing devices. These findings suggest that this new technology may make vector control in HAT foci an affordable option.

## Introduction

African sleeping sickness or Human African Trypanosomiasis (HAT) is endemic to 36 countries in sub-Saharan Africa covering 9 million km^2^ with 60 million of the 400 million inhabitants at risk of the disease. Africa has emerged from a recent sleeping sickness epidemic. In 1997 about 450,000 people were afflicted [1] which has now been reduced to about 70,000 cases per year [2], [3]. Two forms of the disease exists, the Rhodesian (or East African) form being more acute and the Gambian form more chronic. Both these forms of the disease are fatal if left untreated and has an impact of 1.59M DALYs (disability adjusted life years). The related disease (nagana) in domesticated animals causes estimated losses to African agriculture of US$4.5bn per year [4]. In 2000 the African Union recognized trypanosomiasis as “one of Africa's' greatest constraints to socio-economic development” [5]. The trypanosomes causing HAT are transmitted by tsetse flies, particularly those of the Riverine (Palpalis) group. Antigenic variation in the trypanosome makes it unlikely that an effective vaccine will be produced in the foreseeable future. The available drugs are too toxic for prophylactic use. Consequently the only means of preventing the disease is vector control although this is not routinely practiced largely because of the cost.

Drug treatment of HAT is in a parlous state. The drugs available were developed many years ago and their toxicity and consequent human mortality allied to the increasing resistance to the drugs is a great worry [6]. Recent introduction of Nifurtimox Eflornithine Combination Therapy (NECT) has improved the situation but there is serious concern that no other drug for stage II treatment is in reserve should this fail. Vector control is essential for control of the Rhodesiense form of the disease [7] and can play a valuable role in support of case detection and treatment programmes for the Gambiense form of the disease especially in areas of high tsetse challenge when case detection and treatment alone is insufficient for control to be achieved [8], [9]. Given worries about the sustainability of case detection and treatment it is essential that effective vector control measures are available.

A major obstacle in control programmes against Riverine tsetse is cost. Consequently, for the reasons given above, cheaper control techniques are needed. A standard method for control of Riverine tsetse is to use biconical traps, treated or untreated with insecticide or large insecticide-treated targets [9], [10], [11], [12]. Because of their size both are expensive to make and deploy at the high densities required (10–30+/km^2^). Our aim is to develop a more cost-efficient device than the standard biconical trap or 1 m^2^ targets. Work is underway on developing artificial odour attractants to improve device efficiency [13]. Other studies have looked for improvements in the colour and shape of targets and traps [14], [15], [16]. However, few studies have focused on reduction of size of targets as a way to achieve better cost efficiency. Recent work on *G. f. fuscipes* [17] has shown the potential for a dramatic reduction in target size promising a considerable cost saving in control programmes against Riverine tsetse.

Crudely combining data for the number of HAT cases by country [18] and maps of potential distribution of tsetse flies [19] suggests than >90% of current HAT transmission is being caused by a small number of tsetse flies especially *G. fuscipes fuscipes* (Uganda, Sudan, Congo Brazzaville, Central African Republic), *Glossina fuscipes quanzensis* (DRC, Angola, Congo Brazzaville) with smaller number being transmitted by *G. palpalis gambiensis* and *G. p. palpalis* on the coast of West Africa. In this work we have expanded studies on target size to four other species of Riverine tsetse including the very important vectors *G. f. quanzensis* and *G. p. gambiensis*. In addition we have investigated the efficacy of the common practise of insecticide-treating biconical traps in the belief that this increases the number of tsetse they kill beyond those actually trapped by the device [20].

## Methods

### Study sites

We conducted studies in each country during periods considered to be most appropriate in terms of fly abundance, accessibility, time and logistics. In doing so we could not investigate the effect of long-term seasonality on the efficiency of the different devices, nor was this the object of the current study.

Studies were undertaken on *Glossina tachinoides* and *G. palpalis gambiensis* along the lower Comoe river at Folonzo (09° 54′ N, 04° 36′ W) in southern Burkina Faso, between January and May 2009. The two species are sympatric here, along with *G. m. submorsitans* and *G. medicorum*. Additional studies on *G. p. gambiensis* were conducted along the Mouhoun river near Solenzo (12°14′ N, 04°23′ W), in western Burkina Faso, from January to June and in November 2009. See [21] for further details of the site.

Studies were undertaken on *G. fuscipes quanzensis* in July 2009 near the Lukaya river (4° 29′ S, 15° 18′ E), ∼20 km south east of Kinshasa, Democratic Republic of Congo. See [13] for further details of the site.

Studies were undertaken on *G. f. martinii* in November 2009 in the Gombe National Park (4° 38′ S, 29° 37′ E) on the shore of Lake Tanganyika, Tanzania. The area receives an annual rainfall of 760–1200 mm and is in a protected area of tropical rain and highland forest. There are several game species in the research area, including bushpigs (*Potamochoerus porcus*), monitor lizards (*Varanus niloticus*), bushbuck (*Tragelaphus scriptus*), olive baboons (*Papio anubis*), chimpanzees (*Pan troglodytes*) and various species of monkey and snake. *G. brevipalpis* also occurs here.

Studies were performed on *Glossina f. fuscipes* from September to November 2010 on the 0.5 km^2^ of Chamaunga Island (0^0^ 25′ S, 34^0^13′ E), Lake Victoria, Kenya. See [13], [17] for further details of the site.

### Experimental design

**Figure 1:**
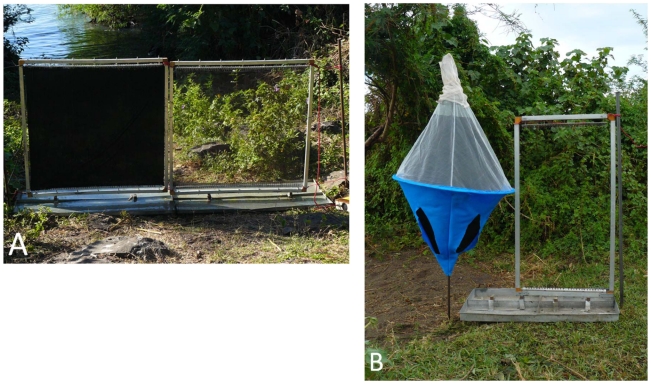
This shows the standard control devices against which new designs were compared. Each is flanked by an electric net to catch flies which circle the device but do not land. Electrified black target with flanking net (A) and a biconical trap with flanking net (B).

Square black targets (1×1 m) were compared for their ability to kill tsetse flies with targets 1/16^th^ the size (0.25×0.25 m) and with a standard biconical trap [12] (Fig. 1). Targets were made from black cotton cloth. Electrocuting grids were fitted over fine black netting and these were placed next to targets and traps where they intercepted flies in flight – these devices are called flanking nets. The fine black polyester net (Quality no. 166, Swisstulle, Nottingham, UK) and the blackened 0.2 mm diameter electrocuting wires of the electric net are effectively invisible to tsetse [22], [23]. Electrocuting grids were also placed over the black cloth target. Electrocuted flies fell into trays of soapy water below the grids. All treatments were simultaneously compared with and without flanking nets [14], [17], allowing us to measure efficiency of the devices (i.e., the catch of the black cloth target or the catch inside the trap as a percentage of the total number of flies arriving in the vicinity of the device). The total number of visiting flies was taken to be the catch in the trap or on the target, plus the catch on each flanking net.

Experiments ran for 12 days each and were carried out during peak activity times of each tsetse species during the period of this study: for *G. tachinoides* and *G. p. gambiensis* from 08:00–12:00; for *G. f. martinii* from 10:00–14:00; for *G. f. quanzensis* from 10:30–14:30. The standard experimental design was a series of Latin-squares of treatments x days x sites, with sites at least 50 m apart. Analyses of variance were performed on log detransformed catches and these are discussed in the text.

**Figure 2:**
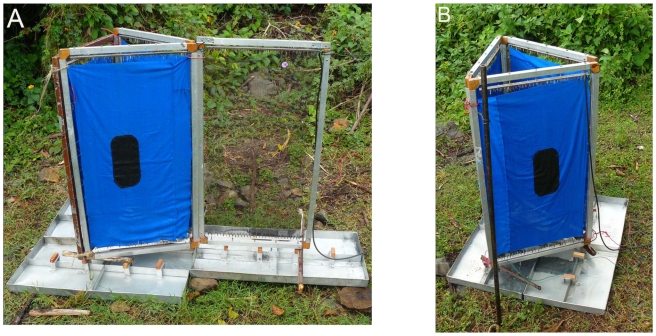
The experimental design used to investigate *G. f. fuscipes* circling or landing on an object. 3-D object with (A) and without (B) flanking net.

Three experiments were conducted to assess the responses of tsetse to 3-dimensional objects. These studies were conducted with *G. f. fuscipes* only. The first experiment measured both the numbers of *G. f. fuscipes* caught on electrified 3-dimensional objects (3DO) (e.g. biconical traps) and the numbers of flies circulating but not contacting such objects. Due to difficulty in covering the conical parts of the biconical trap with electrified grids, a comparable 3 dimensional trap (Fig.2) was made which has flat surfaces. The first experiment compared a fully electrified 3DO consisting of three 0.5×1 m electrified grids arranged in a triangular fashion (Fig. 2) and killing all flies coming into contact with the grid, with a similar 3DO (not electrified) but with an adjacent electrified flanking net which intercepted and killed all circling flies. Each of the grids in the 3DO had a blue cotton cloth insert with a central oblong (15×25 cm) piece of black cloth (to simulate the entrance of a biconical trap). This experiment allowed us to compare numbers of flies attracted to and directly landing on a 3-D object against those flies attracted to, but only circling the object and getting caught on the flanking net. The experiment ran for 12 days in a 2×2 Latin square, from 09:00–12:00.

A second experiment was done with a single flanking net (0.5×1 m) adjacent to a biconical trap to intercept circling flies (Fig 1 image on right), compared against a single biconical trap and against a small blue cotton target (25×25 cm) with an adjacent flanking net (25×25 cm). The small target was also used to compare the efficiency of this small device compared to biconical traps. The reason a blue cotton target was used for these experiments and not black as in the size reduction study, is because blue proved to be a better attractant than black for *G. f. fuscipes* [14], [17] and this type of tiny target is being considered for control purposes.

For the third experiment, we compared a biconical trap closely surrounded with four flanking nets (0.5×1 m) to intercept all flies coming close to the trap as if to land. This was compared against a normal biconical trap as well as a small blue cotton target (25×25 cm) with an adjacent flanking net (25×25 cm). This experiment indicated the number of flies attracted to a biconical trap, but killed on the flanking nets before they could enter or land, compared against the numbers of flies in the top-cage of the standard trap. Again the small target with flanking net was used as control.

Four experiments evaluated the optimal flanking net width for use with a small 25×25 cm target. The first experiment investigated how closely *G. f. fuscipes* circle around a 25×25 cm blue target. This target was used with a 25×100 cm flanking net for 12 days with the collection tray divided into sections 10 cm wide. This determined where flies first touched the flanking net to give an initial indication of the optimal width of a netting panel. Second, we compared flanking nets of 25 cm, 50 cm and 75 cm widths, in a 3×3 Latin square design for 24 days. Third, a 25×25 cm blue target with the same size flanking net was compared with a 12.5×25 cm target with 12.5×25 cm flanking net for 12 days. Finally, a 25×25 cm blue target with 25×25 cm flanking net (all electrified) was compared against an un-electrified 25×25 cm blue target with a 25×25 cm electrified flanking net, in a 2×2 Latin square design for 12 days.

## Results

Catches for all four tsetse species from the devices listed are shown in Table 1. Below we expand and emphasise some of the data which we feel are the most important for the production of more cost effective tsetse killing devices.

**Table 1:** Detransformed mean catches from the experiments to investigate the effect of target size on tsetse fly catches (ANOVA data is given in supplementary Table S1).

Species	Device	Target Size (m^2^)	Flanknet	Males	Flies killed /m^2^	Females	Flies killed/m^2^
*G. tachinoides*							
	Target	2	Yes	27.8a	13.9	28.4a	14.2
	Target	1	No	13.4ab	13.4	9.3b	9.3
	Target	0.125	Yes	11.2b	90.0	6.8b	54.2
	Target	0.0625	No	1.6c	25.1	0.6c	10.2
	Trap	3.5	Yes	50.2ad	14.3	54.2a	15.5
	Trap	3	No	27.2abd	9.1	19.0ab	6.3
			s.e.d.	0.11		0.12	
			P	<0.001		<0.001	
*G. p. gambiensis*							
Folonzo	Target	2	Yes	6.5a	3.2	6.2a	3.1
	Target	1	No	3.1ab	3.1	2.3ab	2.3
	Target	0.125	Yes	3.6abc	28.7	1.9bc	15.0
	Target	0.0625	No	0.4d	6.9	0.3cd	4.9
	Trap	3.5	Yes	7.6ac	2.2	8.1a	2.3
	Trap	3	No	2.0bc	0.7	1.2bcd	0.4
			s.e.d.	0.1		0.12	
			P	<0.001		<0.001	
*G. p. gambiensis*							
Solenzo	Target	2	Yes	3.3abc	1.6	3.6ab	1.8
	Target	1	No	3.7ac	3.7	2.1ab	2.1
	Target	0.125	Yes	2.9abc	23.2	2.3ab	18.2
	Target	0.0625	No	1.2bc	19.4	0.2c	3.0
	Trap	3.5	Yes	5.7a	1.6	5.2ab	1.5
	Trap	3	No	1.8c	0.6	2.3b	0.8
			s.e.d.	0.11		0.1	
			P	<0.001		<0.001	
*G. f. quanzensis*							
	Target	2	Yes	1.9a	0.9	3.2a	1.6
	Target	1	No	1.2ab	1.2	0.8b	0.8
	Target	0.125	Yes	0.4abc	3.5	0.5bc	4.4
	Target	0.0625	No	0.1bcd	2.0	0.1bcd	0.9
	Trap	3.5	Yes	1.8abce	0.5	2.3a	0.7
	Trap	3	No	0.6abcde	0.2	0.5bcd	0.2
			s.e.d.	0.11		0.08	
			P	0.004		<0.001	
*G. f. martinii*							
	Target	2	Yes	0.8a	0.4	0.3a	0.2
	Target	1	No	0.2ab	0.2	0.1ab	0.1
	Target	0.125	Yes	0.5abc	4.4	0.2abc	1.5
	Target	0.0625	No	0.0bcd	0.0	0.0abcd	0.0
	Trap	3.5	Yes	1.1ac	0.3	1.0e	0.3
	Trap	3	No	0.0bcd	0.0	0.0abcd	0.0
			s.e.d.	0.06		0.05	
			P	<0.001		<0.001	

For ease of comparison the efficiency of each device is also expressed in terms of the number of male and female tsetse killed per 1m^2^ of material. Note the absolute numbers of flies caught is merely a reflection of the density of flies in each experimental site; as this varies no comparison between sites can be made. The informative datum is the ratio between flies/m^2^ for each device considering each site separately. Means not associated with the same letter differ at P<0.05. Standard error of differences (s.e.d.) refer to transformed means, which are not shown. See Supplementary data Table S1 for ANOVA tables.

### Large vs small target

The small target with flanking net uses 1/8^th^ of the material in the large 1 m^2^ target. From Table 1 it can be seen that deploying the available cloth in the form of small rather than large targets will kill more tsetse flies per dollar spent. Female flies are the main target of control operations. If we consider just females from Table 1 then we see that for *G. p. gambiensis* the catch per m^2^ for small targets plus flanking nets is between 6.5× (Folonzo), and 8.7× (Solenzo) greater than that for 1 m^2^ targets. Corresponding figures for *G. f. quanzensis* are 5.5×, 5.8× for *G. tachinoides* and 15× for *G. f. martinii,* although in the last case the samples sizes are small. Figures for male flies show even greater potential for small targets. These findings are in agreement with those from a previous study on *G. f. fuscipes* [17].

### Biconical trap vs small target

The small target with flanking net uses 1/24^th^ of the material in the biconical trap. From Table 1 we can see that deploying the available cloth in the form of a small target rather than a biconical trap will kill more tsetse flies per dollar spent. Female flies are the main target of control operations. If we consider just females from Table 1 then we see that for *G. p. gambiensis* the catch per m^2^ for small targets plus flanking nets is between 22.8× (Solenzo) and 37.5× (Folonzo) greater than that for biconical traps. Corresponding figures for *G. f. quanzensis* are 22×, for *G. tachinoides* 8.6× and for *G. f. martinii* it was impossible to determine as the biconical trap failed to catch any flies. Figures for male flies show even greater potential for small targets. Again, these data are in agreement with those from a previous study on *G. f. fuscipes* [17].

### Circling or landing flies

Investigations into the behaviour of *G. f. fuscipes* towards a rectangular blue and black 3-D object showed that 2.6x more *G. f. fuscipes* females circled (mean = 10.9) around the object than landed (mean = 4.2, s.e.d. = 0.09, P = 0.001; for ANOVA see Table S1, experiment 1). For male *G. f. fuscipes* catches of landing flies (mean = 4.5) were roughly equal to the circling flies (mean = 4.9, P = 0.6, s.e.d. = 0.05). When using the biconical trap as a 3-D object the majority of *G. f. fuscipes* circle around the trap but do not enter as can be seen below. Compared to the standard trap, a trap surrounded with four flanking nets caught 4.5x more female *G. f. fuscipes* (mean = 18.1, s.e.d. = 0.11, P<0.001; for ANOVA see Table S1, experiment 2) and a trap with a single adjacent flanking net caught 2.9x more females (mean = 12.2, s.e.d. = 0.06, P<0.001; for ANOVA see Table S1, experiment 3). Male flies also circled more around the trap, with 3.6x more males caught on the single flanking net (mean = 12.2, s.e.d. = 0.06, P = <0.001) and 2.6x more caught on the four flanking nets closely surrounding the trap (mean = 9.2, s.e.d. = 0.1, P<0.001), than were caught inside the standard trap. These data showed that up to 80% of *G. f. fuscipes*, especially females, are circling the trap and not landing or entering giving the biconical trap only about a 20% efficiency.

**Figure 3:**
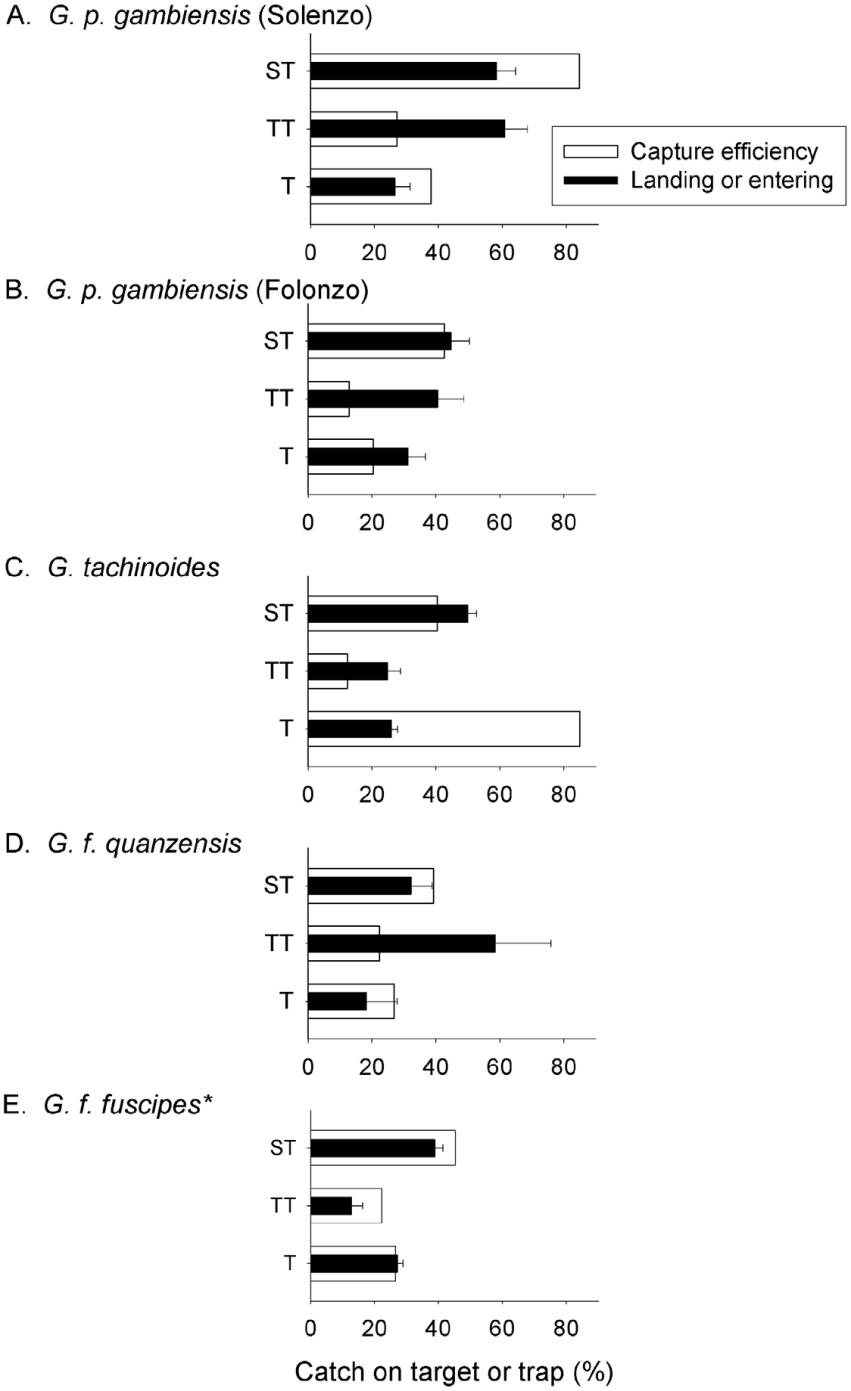
Landing or entering response for tsetse on a standard target 1m^2^ (ST), a small 25×25cm target (TT) or biconical trap (T). For devices with a flanking net, landing or entering responses were estimated by expressing number caught landing on the target or entering the trap as a percentage of the total (device+flanking net) catch. For unaccompanied traps and targets, capture efficiency was estimated by expressing the mean catch of the trap or target as a proportion of the mean catch from a trap+flanking net. A  =  *G. p. gambiensis* at Solenzo; B  =  *G. p. gambiensis* at Folonzo; C  =  *G. tachinoides*; D  =  *G. f. quanzensis*; E  =  *G. f. fuscipes.* *Data for *G. f. fuscipes* derived from Lindh *et al., 2009.*

Comparing the efficiency of the devices for inducing landing and entering responses, the biconical trap again performed poorly (Fig. 3). Only 26% of the *G. tachinoides* and 32% *G. p. gambiensis* attracted to the trap actually entered it. Trap efficiency was even lower for *G. f. quanzensis* (18%), with the majority of flies circling around but not entering. Catches of *G. f. martinii* were too low to allow for analysis of its landing and trap-entry responses. In contrast, the efficiency of the large target (*i.e.* landing response) was much better. Fifty-five percent of *G. tachinoides*, 38% of *G. f. quanzensis* and 45% to 58% of *G. p. gambiensis* that were attracted to the target landed on the black cloth. The small black target with flanking net also induced a poor landing response on the black cloth (Fig. 3), indicating the importance of a flanking net to maintain the killing efficiency of the small target. For example, catches of *G. tachinoides* declined by 88% and *G. f. quanzensis* by 83% in the absence of this netting (*i.e*. catches on the 0.25×0.25 m black target alone), while *G. p. gambiensis* were 50–90% lower without the small flanking net (Table 1).

### Further refinement of the small target

Studies to optimize the flanking net width showed that *G. f. fuscipes* circled closely around the small blue target. Sixty one percent (n = 32, s.e.d. = 0.1) of females and 77% (n = 24, s.e.d. = 0.07) of males were caught on the first 30 cm of flanking net adjacent to the target. A further 23% (n = 12) female and 21% (n = 7) male flies circled up to 50 cm away from the target. The remaining few flies were caught 50–80 cm away, with no flies caught between 80–100 cm. The subsequent experiments with flanking nets of various width showed no difference in catches between the standard 25 cm flanking net (mean = 14.4, sed = 0.05, P = 0.07 for difference between means), the medium 50 cm flanking net (mean = 16.7), or the long 75 cm flank net (mean = 20.3). A smaller flanking net of 12.5×25 cm resulted in a 66% decrease in catches. Equal numbers of flies were caught by the electrified flanking net (mean = 10.4, s.e.d. = 0.09, P = 0.9) adjacent to the un-electrified small blue target, as were caught by the completely electrified target and flank net (mean = 10.2) . This suggests that savings could be made by putting insecticide only on the flanking net.

## Discussion

The catch of tsetse increases with target size but the increase is not in proportion to the increase in surface area. So, paradoxically, it is more cost efficient to deploy the available cloth in the form of small rather than large targets . Tiny targets plus flanking nets use 1/8 and 1/24 the amount of materials required respectively for the large 1 m^2^ targets or biconical traps which are currently used in control programmes. Despite this they are comparable or superior to these much larger devices in killing *G. p. gambiensis*, *G. f. quanzensis, G. f. martini,* (Table 1) and *G. f. fuscipes* [17]. Clearly this means that considerable cost efficiencies are possible in using these new devices as reflected in the tsetse killed per unit area of cloth (Table 1). For example, concentrating only on female tsetse which are the major target of control programmes, the killing effectiveness measured as the catch per m^2^ of cloth for small targets plus flanking nets is 5.5–15× greater than that for 1 m^2^ targets. In comparison to biconical traps, the killing efficiency of small targets plus flanking nets is 8.6–37.5X greater . The tsetse species studied here are responsible for the transmission of virtually all gambiense-form HAT, which represents >90% of all cases of HAT. Hence, the cost savings implied by the above are available to most sleeping sickness control programmes.

Comparison with other tsetse species on which the effects of target size has been studied, is limited to the savannah tsetse. For *G. pallidipes* and *G. morsitans,* a target much less than about 1 m^2^ is strongly contra-indicated [24], [25], [26] due to low attractiveness. This is in strong contrast to our results on Riverine species shown here and in a previous study on *G. f. fuscipes* [17]. The underlying behavioural differences between Riverine and Savanna tsetse which underpin these findings remain to be explained.

An essential part of the small target is the flanking net, as catches of *G. tachinoides*, *G. p. gambiensis* and *G. f. quanzensis* declined by 88%, 67–91% and 83% respectively, in the absence of netting. This illustrates the importance of small panels of fine, insecticide-treated net attached to the side of the small cloth targets to intercept the flies that circle around the cloth. This principle has been used as part of control targets for savannah species [24] and recommended for control of *G. p. gambiensis* and *G. tachinoides* [18]. However, large panels of netting are prone to damage which renders large 1 m^2^ devices fixed with a netting panel inefficient. With the tiny targets recommended by this work, the small flanking net is much less likely to be damaged. In addition, suitable netting now available on the market, particularly insecticide pre-impregnated polyethylene netting, is stronger and more durable.

A common practice in the control community has been to use insecticide-treated traps in the belief that many more flies will land on the outside of traps than are caught by them [27]. However, there are scant direct data supporting this practice and hence it is not universally accepted. Observations of *G. morsitans* and *G. pallidipes* showed that only 47–30% of tsetse approaching a trap landed on it or entered it, i.e.the majority (53–70%) of tsetse visiting a trap did not contact it [28]. Our data show that the efficiency (proportion of the total flies attracted to the trap which are actually caught by it) is low (e.g. 26% *G. tachinoides*; 32% *G. p. gambiensis*; 10% *G. f. quanzensis*). Let us assume for the sake of argument that 100% of the flies circulating the biconical trap in our experiments land on it and collect a lethal dose of insecticide. Even then, using the data from Table 1, the flies killed per 1 m^2^ of cloth will be greater for small targets plus a flanking net than for biconical traps (2.2X *G. tachinoides*; 12X and 7.3X *G. p. gambiensis*; 2.2X *G. f.* quanzensis). In fact, the results show that the catch from the 3-D target with a flanking net was 1.8x that of the target alone (15.9 tsetse/day vs. 8.7 tsetse/day) suggesting that not all tsetse approaching the object landed on it. The efficiency of the trap-like object (55%) is slightly greater than a trap (31%) suggesting that marginally more flies may land on a trap than are captured by it. If that figure is common to all species it would roughly double the kill per m^2^ figures given above in this paragraph. Clearly small targets plus flanking nets are a more efficient means of killing tsetse than using either 1 m^2^ targets or biconical traps whether the latter are treated with insecticide or not.

This work clearly demonstrates the potential savings for tsetse control operations in terms of reduced costs of materials and insecticide associated with the manufacture of small targets. In addition these devices are likely to offer two further advantages. First, the small targets would be considerably easier and cheaper to transport to the field [28] offering further considerable cost savings to control campaigns. For example, the tiny targets can be carried in a backpack and deployed rapidly by a single person. Second, while large targets and their associated doses of insecticide have been shown to have little impact on ecology [29] and to be unobtrusive in national parks [30], the small targets could be expected to be even better in both these respects. A potential problem for small targets is that they may be easily obscured by vegetation which may reduce their efficiency. Further work is underway to look at the importance of this and the indications are that this is very much smaller problem for Palpalis group flies than for Morsitans group flies (Esterhuizen et al., in preparation).

In conclusion, it appears that the use of small targets demands a full scale field trial while further research should be performed to refine them and to explore their applicability against a wider range of tsetse species and in other areas.

## Supporting Information

Table S1 ANOVA tables for log-transformed catches of male and female tsetse from targets (see Table 1).
(DOCX)
