# Supplementary material for: Improving the Cost-Effectiveness of Visual Devices for the Control of Riverine Tsetse Flies, the Major Vectors of Human African Trypanosomiasis
Source: PLoS Negl Trop Dis. 2011 Aug 2;5(8):e1257. doi: 10.1371/journal.pntd.0001257 (PMC3149014; doi:10.1371/journal.pntd.0001257)
Supplement: Table S1 — ANOVA tables for log-transformed catches of male and female tsetse from targets (see Table 1). (DOCX) [file pntd.0001257.s001.docx]

|  |  |  | Males | | | Females | | |
| --- | --- | --- | --- | --- | --- | --- | --- | --- |
| Species | Source of variation | df | SS | MS | F | SS | MS | F |
| *G. tachinoides* | Day | 11 | 2.81 | 0.26 | 3.8 | 1.64 | 0.15 | 1.8 |
|  | Site | 5 | 0.68 | 0.14 | 2.0 | 1.21 | 0.24 | 2.9 |
|  | Treatment | 5 | 12.33 | 2.47 | 36.5*** | 17.07 | 3.41 | 40.5*** |
|  | Residual | 50 | 3.38 | 0.07 |  | 4.21 | 0.08 |  |
|  | Total | 71 | 2.81 | 0.26 |  | 24.12 |  |  |
| *G. p. gambiensis (F)* | Day | 11 | 2.43 | 0.22 | 3.7 | 1.91 | 0.17 | 2.1 |
|  | Site | 5 | 2.68 | 0.54 | 8.9 | 1.10 | 0.22 | 2.6 |
|  | Treatment | 5 | 4.84 | 0.97 | 16.1*** | 6.06 | 1.21 | 14.5*** |
|  | Residual | 50 | 3.01 | 0.06 |  | 4.19 | 0.08 |  |
|  | Total | 71 | 12.95 |  |  | 13.26 |  |  |
| *G. p. gambiensis (S)* | Day | 11 | 0.91 | 0.08 | 1.2 | 1.75 | 0.16 | 4.1 |
|  | Site | 5 | 0.83 | 0.17 | 2.4 | 0.60 | 0.12 | 3.1 |
|  | Treatment | 5 | 1.77 | 0.35 | 5.1*** | 3.51 | 0.70 | 18.2*** |
|  | Residual | 50 | 3.50 | 0.07 |  | 1.93 | 0.04 |  |
|  | Total | 71 | 7.01 |  |  | 7.78 |  |  |
| *G. quanzensis* | Day | 11 | 1.51 | 0.14 | 1.7 | 1.23 | 0.11 | 2.5 |
|  | Site | 5 | 3.04 | 0.61 | 7.5 | 2.09 | 0.42 | 9.2 |
|  | Treatment | 5 | 1.65 | 0.33 | 4.1** | 3.12 | 0.62 | 13.7*** |
|  | Residual | 50 | 4.08 | 0.08 |  | 2.27 | 0.05 |  |
|  | Total | 71 | 10.27 |  |  | 8.71 |  |  |
| *G. martinii* | Day | 11 | 0.80 | 0.07 | 2.9 | 0.11 | 0.01 | 0.6 |
|  | Site | 5 | 0.64 | 0.13 | 5.1 | 0.16 | 0.03 | 1.9 |
|  | Treatment | 5 | 1.11 | 0.22 | 9.0*** | 0.76 | 0.15 | 9.3*** |
|  | Residual | 50 | 1.24 | 0.02 |  | 0.82 | 0.02 |  |
|  | Total | 71 | 3.78 |  |  | 1.84 |  |  |

Significant effects are shown for Treatments only at the *P*<0.001 (***), *P*<0.01 (**) levels of probability. Studies of *G. p. gambiensis* were conducted in Folonzo (F) and Solenzo (S).
